# Supplementary material for: Identification of Freezing-Responsive microRNAs and Their Targets in Chinese Jujube by Small RNA and Degradome Sequencing
Source: Int J Mol Sci. 2026 Sep 4;27(17):7912. doi: 10.3390/ijms27177912 (PMC13566890; doi:10.3390/ijms27177912)
Supplement: Supplementary file 1 [file ijms-27-07912-s001.zip › Supplementary Material S2/novel_miR_220.pdf]

Provisional ID	:	novel_miR_220
Score total	:	13112.4
Score for star read(s)	:	3.9
Score for read counts	:	13106.2
Score for mfe	:	0.7
Score for randfold	:	1.6
Score for cons. seed	:	
Total read count	:	25719
Mature read count	:	23969
Loop read count	:	6
Star read count	:	1744

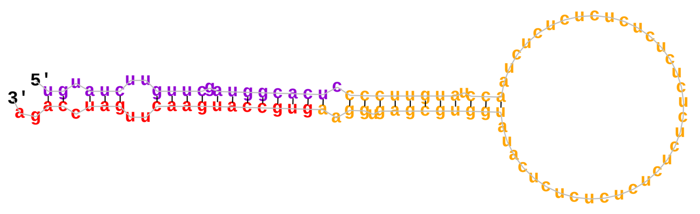

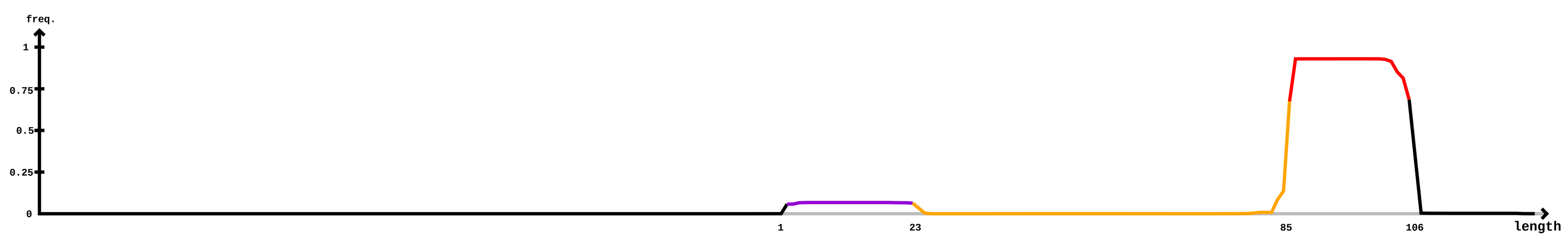


```
                                                                                                                                  Star                                                                     Mature
5'- caauuagguuuagugaacagaagugccuuuuuuuuuuuuuuuuuuuggguuaggaugcgacuauaaaaagcagaggauggugggagaagaaauacgccacuuccaucuuguucguucacccucuguaucuuguucgauggcacuccccuuguauccaaucucucucucucucucucucucucucucucauauggugcgaguggaagugccaugaacuugauccagaaggugcucucgaacaaguuaca  -3'  obs
    caauuagguuuagugaacagaagugccuuuuuuuuuuuuuuuuuuuggguuaggaugcgacuauaaaaagcagaggauggugggagaagaaauacgccacuuccaucuuguucguucacccucuguaucuuguucgauggcacuccccuuguauccaaucucucucucucucucucucucucucucucauauggugcgaguggaagugccaugaacuugauccagaaggugcucucgaacaaguuaca       exp
    .......((((...)))).(((((((.(((((((((((..(((((((...(((.......))).......)))))))....)))))))))))...).))))))...((((((((..((((.((((.(((..((((.((((((((.((((((((.(((..............................))))))))).)).)))))))))))..))).)))).))))....))))))))....  reads  mm  sample
    ...............................................gguuaggaugcgacuauaaaaGgcagagg..............................................................................................................................................................................  1  1  JCK-3
    .............................................................................................acgccacuuccaucuuguucg........................................................................................................................................  1  0  LT-1
    .......................................................................................................................ccucuguaucuuguucgauggc.............................................................................................................  2  0  JCK-2
    .......................................................................................................................ccucuguaucuuguucgauggc.............................................................................................................  1  0  JCK-1
    ........................................................................................................................cucuguaucuuguucgauggc.............................................................................................................  2  0  JCK-2
    ...........................................................................................................................uguaucuuguucgauggc.............................................................................................................  1  0  LT-2
    ...........................................................................................................................ugCaucuuguucgauggc.............................................................................................................  2  1  LT-2
    ...........................................................................................................................ugNaucuuguucgauggc.............................................................................................................  13  1  LT-2
    ...........................................................................................................................ugAaucuuguucgauggc.............................................................................................................  1  1  LT-1
    ...........................................................................................................................ugAaucuuguucgauggc.............................................................................................................  1  1  LT-3
    ...........................................................................................................................ugAaucuuguucgauggc.............................................................................................................  1  1  LT-2
    ...........................................................................................................................ugAaucuuguucgauggc.............................................................................................................  1  1  LCK-2
    ...........................................................................................................................ugAaucuuguucgauggc.............................................................................................................  1  1  LCK-3
    ...........................................................................................................................ugAaucuuguucgauggca............................................................................................................  1  1  LCK-3
    ...........................................................................................................................ugAaucuuguucgauggca............................................................................................................  1  1  LT-2
    ...........................................................................................................................ugAaucuuguucgauggca............................................................................................................  2  1  LCK-1
    ...........................................................................................................................ugNaucuuguucgauggca............................................................................................................  1  1  LT-2
    ...........................................................................................................................ugAaucuuguucgauggca............................................................................................................  1  1  LCK-2
    ...........................................................................................................................ugNaucuuguucgauggcac...........................................................................................................  4  1  LT-2
    ...........................................................................................................................ugAaucuuguucgauggcac...........................................................................................................  1  1  JCK-3
    ...........................................................................................................................ugAaucuuguucgauggcacu..........................................................................................................  1  1  JCK-1
    ...........................................................................................................................ugAaucuuguucgauggcacu..........................................................................................................  3  1  LCK-3
    ...........................................................................................................................ugCaucuuguucgauggcacu..........................................................................................................  1  1  LT-2
    ...........................................................................................................................ugNaucuuguucgauggcacu..........................................................................................................  8  1  LT-2
    ...........................................................................................................................ugAaucuuguucgauggcacu..........................................................................................................  3  1  JCK-3
    ...........................................................................................................................ugAaucuuguucgauggcacu..........................................................................................................  4  1  LT-2
    ...........................................................................................................................uguaucuuguucgauggcacu..........................................................................................................  6  0  JCK-3
    ...........................................................................................................................Cguaucuuguucgauggcacu..........................................................................................................  1  1  JCK-3
    ...........................................................................................................................ugAaucuuguucgauggcacu..........................................................................................................  2  1  LT-3
    ...........................................................................................................................uguaucuuguucgauggcacu..........................................................................................................  15  0  JCK-2
    ...........................................................................................................................uguaucuuguucgauggcacu..........................................................................................................  1  0  LCK-3
    ...........................................................................................................................ugAaucuuguucgauggcacu..........................................................................................................  8  1  JCK-2
    ...........................................................................................................................ugAaucuuguucgauggcacu..........................................................................................................  3  1  LT-1
```

Star Mature

```
caauuagguuuagugaacagaagugccuuuuuuuuuuuuuuuuuuuuggguuaggaugcgacuauaaaaagcagaggauggugggagaagaaauacgccacuuccaucuuguucguucacccucuguaucuuguucgauggcacuccccuuguauccaaucucucucucucucucucucucucucucucucucauauggugcgaguggaagugccaugaacuugauccagaaggugcucucgaacaaguuaca
............................................................................................................................ugNaucuuguucgauggcacu............................................................................................................	1	1	LCK-1
............................................................................................................................uguaucuuguucgauggcacu............................................................................................................	6	0	JCK-1
............................................................................................................................uguauUuuguucgauggcacuc...........................................................................................................	1	1	JCK-1
............................................................................................................................uNuaucuuguucgauggcacuc...........................................................................................................	1	1	JCK-3
............................................................................................................................ugCaucuuguucgauggcacuc...........................................................................................................	1	1	LT-2
............................................................................................................................ugAaucuuguucgauggcacuc...........................................................................................................	2	1	LT-1
............................................................................................................................uguaucuuguucgauggcacuc...........................................................................................................	4	0	JT-2
............................................................................................................................uguaucuuguucgauggcacuc...........................................................................................................	23	0	JT-1
............................................................................................................................ugAaucuuguucgauggcacuc...........................................................................................................	2	1	LT-2
............................................................................................................................uguaucuuguucgauggcacuc...........................................................................................................	312	0	JCK-1
............................................................................................................................ugAaucuuguucgauggcacuc...........................................................................................................	3	1	JCK-1
............................................................................................................................uguaucCuguucgauggcacuc...........................................................................................................	1	1	JCK-2
............................................................................................................................ugAaucuuguucgauggcacuc...........................................................................................................	1	1	LT-3
............................................................................................................................ugNaucuuguucgauggcacuc...........................................................................................................	10	1	LT-2
............................................................................................................................uguaucuuguucgauggcacuc...........................................................................................................	8	0	JT-3
............................................................................................................................uguaucuuguucAauggcacuc...........................................................................................................	1	1	JCK-2
............................................................................................................................uguaucuuguucgauggcacuc...........................................................................................................	425	0	JCK-3
............................................................................................................................uguaucuuguucgauggcacuc...........................................................................................................	555	0	JCK-2
............................................................................................................................uguaucuuguucgCuggcacuc...........................................................................................................	2	1	JCK-2
............................................................................................................................uAuaucuuguucgauggcacuc...........................................................................................................	1	1	JCK-3
............................................................................................................................uguauUuuguucgauggcacuc...........................................................................................................	3	1	JCK-3
............................................................................................................................uguaucuuguucgauggcacuG...........................................................................................................	1	1	JCK-1
............................................................................................................................ugAaucuuguucgauggcacuc...........................................................................................................	1	1	LCK-1
............................................................................................................................uguaucuuAuucgauggcacuc...........................................................................................................	1	1	JCK-2
............................................................................................................................uNuaucuuguucgauggcacuc...........................................................................................................	1	1	JCK-1
............................................................................................................................ugAaucuuguucgauggcacuc...........................................................................................................	3	1	JCK-2
............................................................................................................................uguaucuuguucgauggcCcuc...........................................................................................................	2	1	JCK-2
............................................................................................................................ugAaucuuguucgauggcacuc...........................................................................................................	4	1	JCK-3
............................................................................................................................uguCucuuguucgauggcacuc...........................................................................................................	1	1	JCK-1
............................................................................................................................ugAaucuuguucgauggcacuc...........................................................................................................	1	1	LCK-3
............................................................................................................................uguauUuuguucgauggcacuc...........................................................................................................	1	1	JCK-2
............................................................................................................................uguaucuuguucgauggcacuc...........................................................................................................	2	0	LT-1
............................................................................................................................uguaucuuguucgauggcacucU..........................................................................................................	5	1	JCK-2
............................................................................................................................uguaucuuguucgauggcacucA..........................................................................................................	1	1	JCK-2
............................................................................................................................uguaucuuguucgauggcacucU..........................................................................................................	4	1	JCK-3
............................................................................................................................uguaucuuguucgauggcacucc..........................................................................................................	2	0	JCK-3
............................................................................................................................uguaucuuguucgauggcacucc..........................................................................................................	5	0	JCK-1
............................................................................................................................uguaucuuguucgauggcacucU..........................................................................................................	3	1	JCK-1
............................................................................................................................uguaucuuguucgauggcacucA..........................................................................................................	2	1	JCK-1
............................................................................................................................uguaucuuguucgauggcacucc..........................................................................................................	2	0	JCK-2
.............................................................................................................................guaucuuguucgauggcacucc..........................................................................................................	2	0	JCK-1
..............................................................................................................................uaucuuguucgauggcacuc...........................................................................................................	1	0	JCK-2
..............................................................................................................................uaucuuguucgauggcacucc..........................................................................................................	8	0	JT-1
..............................................................................................................................uaucuuguucgGuggcacucc..........................................................................................................	1	1	JT-1
..............................................................................................................................uaucuuguucgauggcacucU..........................................................................................................	2	1	JCK-3
..............................................................................................................................uaucuuguucgauggcacucA..........................................................................................................	2	1	JCK-1
..............................................................................................................................uaucuuguucgauggcacucU..........................................................................................................	1	1	JCK-2
..............................................................................................................................uaucuuguucgauggcacucc..........................................................................................................	36	0	JCK-3
..............................................................................................................................uaucuuguucgauggcacucc..........................................................................................................	47	0	JCK-2
..............................................................................................................................uaucuuguucgauggcacucA..........................................................................................................	1	1	JCK-2
..............................................................................................................................uaucuuguucgauggcacucU..........................................................................................................	8	1	JT-1
..............................................................................................................................uaucuuguucgauggcacucA..........................................................................................................	2	1	JCK-3
..............................................................................................................................uaucuuguucgauggcacucc..........................................................................................................	27	0	JCK-1
..............................................................................................................................uaucuuguucgauggcacuccc.........................................................................................................	32	0	JCK-2
..............................................................................................................................Aaucuuguucgauggcacuccc.........................................................................................................	1	1	JCK-2
..............................................................................................................................uaucuuguucgCuggcacuccc.........................................................................................................	1	1	JCK-3
..............................................................................................................................uaucuuguucgCuggcacuccc.........................................................................................................	1	1	JCK-2
..............................................................................................................................uaucuuguucgauggcacuccc.........................................................................................................	16	0	JCK-1
..............................................................................................................................uaucuuguucgauggcacuccc.........................................................................................................	25	0	JCK-3
...............................................................................................................................aucuuguucgauggcacu............................................................................................................	1	0	LT-2
...............................................................................................................................aucuuguucgauggcacuc...........................................................................................................	1	0	JCK-3
...............................................................................................................................aucuuguucgauggcacuc...........................................................................................................	1	0	JCK-1
...............................................................................................................................aucuuguucgauggcacuc...........................................................................................................	3	0	LT-3
...............................................................................................................................aucuuguucgauggcacucc..........................................................................................................	3	0	LT-3
...............................................................................................................................aucuuguucgauggcacucU..........................................................................................................	1	1	LT-1
...............................................................................................................................aucuuguucgauggcacucc..........................................................................................................	1	0	JCK-3
...............................................................................................................................aucuuguucgauggcacucc..........................................................................................................	1	0	JCK-2
...............................................................................................................................aucuuguucgauggcacucU..........................................................................................................	2	1	JCK-2
...............................................................................................................................aucuuguucgauggcacucc..........................................................................................................	2	0	LT-2
...............................................................................................................................aucuuguucgauggcacucc..........................................................................................................	1	0	JCK-1
```

Star   Mature

caauuagguuuagugaacagaagugccuuuuuuuuuuuuuuuuuuuuggguuaggaugcgacuauaaaaagcagaggauggugggagaagaaauacgccacuuccaucuuguucguucacccucuguaucuuguucgauggcacuccccuuguauccaaucucucucucucucucucucucucucucucucucauauggugcgaguggaagugccaugaacuugauccagaaggugcucucgaacaaguuaca

aucuuguucgauggcacucU	1	1	JCK-3
aucuuguucgauggcacuccc	1	0	LT-1
aucuuguucgauggcacuccc	1	0	LT-3
aucCuguucgauggcacuccc	1	1	LT-3
aucuuguucgauggcacuccc	2	0	JCK-3
aucuuguucgauggcacuccc	5	0	JCK-2
aucuuguucgauggcacucccc	2	0	LT-3
ucuuguucgauggcacuc	1	0	JCK-3
ucuuguucgauggcacucc	4	0	LCK-3
ucuuguucgauggcacuccc	2	0	LT-3
aucAaaucucucucucucucucucuc	1	1	LT-1
Accaaucucucucucucuc	1	1	LT-3
aucucucucucucuGucuc	1	1	LT-3
ucucucucucuNucucucucucuc	4	1	JCK-1
ucucucucucuNucucucucucuc	4	1	JCK-1
ucucucucucuNucucucucucuc	4	1	JCK-1
ucucucucucuNucucucucucuc	4	1	JCK-1
cucucucucucucuGucuca	1	1	LT-3
ucucucucucGcucucuca	1	1	JCK-1
uggugcgaguggaagugccaugaacu	1	0	LT-3
gugcgaguggaagugccaugaacuug	5	0	LCK-3
gcgaguggaagugccaugaacuugau	2	0	LCK-3
cgaguggaagugccaugaacuugauccagC	1	1	LT-1
cgaguggaagugccaugaacuugauccaga	1	0	LT-1
gaguggaagugccaugaacuugaucc	1	0	JCK-3
gaguggaagugccaugaacuugaucc	1	0	JCK-2
gaguggaagugccaugaacuugauccaga	1	0	JCK-2
gaguggaagugccaugaacuugauccaga	2	0	JCK-3
gaguggaagugccaugaacuugauccaga	1	0	JCK-1
gaguggaagugccaugaacuugauccagaa	1	0	JCK-3
gaguggaagugccaugaacuugauccagaa	1	0	JCK-1
aguggaagugccaugaacuuga	1	0	LT-3
aguggaagugccaugaacuugauc	1	0	JCK-2
aguggaagugccaugaacuugauc	1	0	JCK-1
aguggaagugccaugaacuugauccaga	4	0	JCK-2
aguggaagugccaugaacuugauccaga	1	0	JCK-3
aguggaagugccaugaacuugauccaga	2	0	LCK-3
guggaagugccaugaacuugaucc	1	0	JCK-1
guggaagugccaugaacuugaucc	4	0	JT-3
guggaagugccaugaacuugaucc	2	0	JCK-3
guggaagugccaugaacuugaucc	2	0	JT-2
guggaagugccaugaacuugauccaU	3	1	LCK-3
guggaagugccaugaacuugauccaga	17	0	LCK-2
guggaagugccaugaacuugauccagU	4	1	JCK-1
guggaagugccaugaacuugauccagU	1	1	JCK-2
guggaagugccaugaacuugauccaga	1	0	LT-2
guggaagugccaugaacuugauccagU	1	1	LCK-3
guggaagugccaugaacuugauccaga	1	0	JCK-3
guggaagugccaugaacuugauccagU	1	1	JCK-3
guggaagugccaugaacuugauccaga	4	0	JCK-2
guggaagugccaugaacuugauccaga	27	0	LCK-1
uggaaUugccaugaacuuga	1	1	JCK-3
uggaaUugccaugaacuuga	1	1	JCK-2
uggaaUugccaugaacuugau	2	1	JCK-2
uggaagugccaugaacuugau	2	0	LT-3
uggaaUugccaugaacuugau	1	1	JCK-1
uggaaUugccaugaacuugau	3	1	JCK-3
uggaagugccaugaacuugauc	1	0	JCK-1
uggaagugccaugaacuugauc	4	0	JCK-3
uggaagugccaugaacuugauc	2	0	JCK-2
uggaagugccaugaacuugauU	1	1	JCK-3
uggaagugccaugaacuugauA	1	1	LT-1
uggaagugccaugaacuugauc	1	0	LT-2
uggaaUugccaugaacuugauc	3	1	JCK-1
uggaagugccaugaacuugauU	1	1	JCK-2
uggaaUugccaugaacuugauc	7	1	JCK-2
uggaagugccaugaacuugauU	2	1	LT-3
uggaaUugccaugaacuugaucc	21	1	JCK-2
uggaagugccaugaacuugaucc	5	0	LCK-3
uggaaUugccaugaacuugaucc	2	1	JT-2

```
                                                                                                                              Star                                                                      Mature
caauuagguuuagugaacagaagugccuuuuuuuuuuuuuuuuuuuuuugggguuaggaugcgacuauaaaaagcagaggauggugggagaagaaauacgccacuuccaucuuguucguucacccucuguaucuuguucgauggcacuccccuuguauccaaucucucucucucucucucucucucucucucucucucucauauggugcgaguggaagugccaugaacuugauccagaaggugcucucgaacaaguuaca
................................................................................................................................................................................................................uggaagugccaugaacuugaucc.........................   3   0   LT-1
................................................................................................................................................................................................................uggaagugccaugaacuugaucc.........................   2   0   LT-2
................................................................................................................................................................................................................uggaaUugccaugaacuugaucc.........................   4   1   JCK-1
................................................................................................................................................................................................................uggaagugccaugaacuugaucc.........................   3   0   JCK-1
................................................................................................................................................................................................................uggaagugccaugaacuugaucc.........................   7   0   JCK-2
................................................................................................................................................................................................................uggaagugccaugaacuugaucc.........................   3   0   LT-3
................................................................................................................................................................................................................uggaaUugccaugaacuugaucc.........................   2   1   JT-3
................................................................................................................................................................................................................uggaagugccaugaacuugaucc.........................   2   0   JCK-3
................................................................................................................................................................................................................uggaaUugccaugaacuugaucc.........................   6   1   JCK-3
................................................................................................................................................................................................................uggaaUugccaugaacuugaucca........................   8   1   JCK-2
................................................................................................................................................................................................................uggaaUugccaugaacuugaucca........................   8   1   JCK-3
................................................................................................................................................................................................................uggaaUugccaugaacuugaucca........................   3   1   JCK-1
................................................................................................................................................................................................................uggaagugccaugaacuugauccagC......................   2   1   LT-2
................................................................................................................................................................................................................uggaagugccaugaacuugauccagU......................   1   1   LT-3
..................................................................................................................................................................................................................gaagugccaugaacuugauccaA.......................   1   1   LT-1
...................................................................................................................................................................................................................aagugccaugaacuugUu...........................   1   1   LT-1
...................................................................................................................................................................................................................aagugccaugaacuugau...........................   5   0   JCK-3
...................................................................................................................................................................................................................aagugccaugaacuugUu...........................   2   1   LT-3
...................................................................................................................................................................................................................aagugccaugaacuugaA...........................   2   1   LT-3
...................................................................................................................................................................................................................aagugccaugaacuugau...........................   3   0   LT-2
...................................................................................................................................................................................................................aagugccaugaacuugau...........................   6   0   JCK-2
...................................................................................................................................................................................................................aaUugccaugaacuugau...........................   3   1   JCK-1
...................................................................................................................................................................................................................aagugccaugaacuuUau...........................   4   1   LCK-3
...................................................................................................................................................................................................................aaUugccaugaacuugau...........................   9   1   JCK-2
...................................................................................................................................................................................................................aaUugccaugaacuugau...........................   19  1   JT-1
...................................................................................................................................................................................................................aagugccaugaacuugau...........................   5   0   LT-3
...................................................................................................................................................................................................................aagugccaugaacuugau...........................   2   0   JT-3
...................................................................................................................................................................................................................aagugccaugaacuugau...........................   7   0   LT-1
...................................................................................................................................................................................................................aaUugccaugaacuugau...........................   1   1   JT-2
...................................................................................................................................................................................................................aagugccaugaacuugau...........................   2   0   JCK-1
...................................................................................................................................................................................................................aaUugccaugaacuugau...........................   2   1   JCK-3
...................................................................................................................................................................................................................aaUugccaugaacuugau...........................   3   1   JT-3
...................................................................................................................................................................................................................aagugccaugaacuugauc..........................   1   0   LT-1
...................................................................................................................................................................................................................aagugccaugaacuugauc..........................   22  0   JCK-1
...................................................................................................................................................................................................................aaUugccaugaacuugauc..........................   6   1   JCK-1
...................................................................................................................................................................................................................aagugccaugaacuugauU..........................   5   1   LT-3
...................................................................................................................................................................................................................aaUugccaugaacuugauc..........................   26  1   JT-3
...................................................................................................................................................................................................................aaUugccaugaacuugauc..........................   28  1   JT-1
...................................................................................................................................................................................................................aagugccaugaacuugauc..........................   8   0   LT-3
...................................................................................................................................................................................................................aagugccaugaacuugauU..........................   4   1   JCK-3
...................................................................................................................................................................................................................aaUugccaugaacuugauc..........................   12  1   JCK-3
...................................................................................................................................................................................................................aagugccaugaacuugauU..........................   2   1   JCK-1
...................................................................................................................................................................................................................aaUugccaugaacuugauc..........................   15  1   JCK-2
...................................................................................................................................................................................................................aagugccaugaacuugauc..........................   23  0   JCK-3
...................................................................................................................................................................................................................aagugccaugaacuugauU..........................   1   1   JCK-2
...................................................................................................................................................................................................................aagugccaugaacuugauc..........................   1   0   LT-2
...................................................................................................................................................................................................................aagugccaugaacuugauc..........................   15  0   JCK-2
...................................................................................................................................................................................................................aCgugccaugaacuugauc..........................   1   1   LT-2
...................................................................................................................................................................................................................aagugccaugaacuugauc..........................   8   0   JT-3
...................................................................................................................................................................................................................aagugccaugaacuugauc..........................   3   0   JT-2
...................................................................................................................................................................................................................aagugccaugaacuugauU..........................   2   1   LT-2
...................................................................................................................................................................................................................aagugccaugaacuugauU..........................   6   1   LT-1
...................................................................................................................................................................................................................aagugccaugCacuugauc..........................   1   1   JCK-1
...................................................................................................................................................................................................................aagugccaugaacuugauA..........................   1   1   LT-3
...................................................................................................................................................................................................................aaUugccaugaacuugauc..........................   21  1   JT-2
...................................................................................................................................................................................................................aagugccaugaacuugaucU.........................   3   1   JCK-2
...................................................................................................................................................................................................................aagugccaugaacuugaucU.........................   3   1   JCK-3
...................................................................................................................................................................................................................aagugccaugaacuugaucU.........................   3   1   LT-2
...................................................................................................................................................................................................................aagugccaugaacuugaucc.........................   105 0   JCK-3
...................................................................................................................................................................................................................aaUugccaugaacuugaucc.........................   41  1   JCK-2
...................................................................................................................................................................................................................aagugccaugaacuugaucc.........................   89  0   LCK-3
...................................................................................................................................................................................................................aagugccaugGacuugaucc.........................   1   1   LT-3
...................................................................................................................................................................................................................aagugccaugaacuugaucc.........................   108 0   JCK-2
...................................................................................................................................................................................................................aaUugccaugaacuugaucc.........................   20  1   JT-2
...................................................................................................................................................................................................................aagugccaugaacuugaucU.........................   2   1   LT-1
...................................................................................................................................................................................................................aagugccaugaacuugaucc.........................   93  0   LT-3
...................................................................................................................................................................................................................aagugccaugaacuugaucc.........................   21  0   LCK-2
...................................................................................................................................................................................................................aagugccaugaacuugaucc.........................   62  0   JCK-1
...................................................................................................................................................................................................................aCgugccaugaacuugaucc.........................   1   1   LT-2
...................................................................................................................................................................................................................aagugccaugaacuugaucA.........................   3   1   JCK-3
```

```
                                                                                                                         Star                                                                              Mature
caauuagguuuagugaacagaagugccuuuuuuuuuuuuuuuuuuuggguuaggaugcgacuauaaaaagcagaggaugguggggagaagaaauacgccacuuccaucuuguucguucacccucuguaucuuguucgauggcacuccccuuguauccaaucucucucucucucucucucucucucucucauauggugcgaguggaagugccaugaacuugauccagaaggugcucucgaacaaguuaca
..............................................................................................................................................................................................................aCgugccaugaacuugaucc.........................   1     1    JCK-3
..............................................................................................................................................................................................................aaUugccaugaacuugaucc.........................   26    1    JCK-1
..............................................................................................................................................................................................................aagugccaugaCcuugaucc.........................   1     1    JCK-2
..............................................................................................................................................................................................................aagugccaugaacuugaucA.........................   2     1    JCK-1
..............................................................................................................................................................................................................aagugccaugaacuugaucU.........................   6     1    LCK-3
..............................................................................................................................................................................................................aagugccaugCacuugaucc.........................   1     1    LT-1
..............................................................................................................................................................................................................aagugccaugaacuugaucc.........................   64    0    LT-2
..............................................................................................................................................................................................................aagugcAaugaacuugaucc.........................   1     1    JCK-2
..............................................................................................................................................................................................................aagugcAaugaacuugaucc.........................   1     1    JCK-3
..............................................................................................................................................................................................................aagugccaugaacuugaucc.........................   51    0    LT-1
..............................................................................................................................................................................................................aaUugccaugaacuugaucc.........................   25    1    JCK-3
..............................................................................................................................................................................................................Cagugccaugaacuugaucc.........................   1     1    LT-2
..............................................................................................................................................................................................................aNgugccaugaacuugaucc.........................   1     1    JCK-3
..............................................................................................................................................................................................................aagugccaugaacuugaucU.........................   1     1    LT-3
..............................................................................................................................................................................................................aaUugccaugaacuugaucc.........................   21    1    JT-3
..............................................................................................................................................................................................................aagugccaugaacuugaucU.........................   1     1    JCK-1
..............................................................................................................................................................................................................aagugccaugaacuugaucc.........................   17    0    LCK-1
..............................................................................................................................................................................................................aagugccaugaacuugaucca........................   33    0    LT-2
..............................................................................................................................................................................................................aagugccCugaacuugaucca........................   1     1    JCK-3
..............................................................................................................................................................................................................aagugccaugaacuugaucca........................   120   0    JCK-3
..............................................................................................................................................................................................................aagugccaugaacuugauccC........................   2     1    LT-3
..............................................................................................................................................................................................................aagugccaugaacuugaucca........................   33    0    LT-1
..............................................................................................................................................................................................................aagugccaugaacuugauccC........................   2     1    JCK-3
..............................................................................................................................................................................................................aagugccaugaGcuugaucca........................   1     1    JCK-1
..............................................................................................................................................................................................................aagugUcaugaacuugaucca........................   1     1    JCK-3
..............................................................................................................................................................................................................aagugccaugaacuugaucca........................   93    0    JCK-1
..............................................................................................................................................................................................................aagugccaugaacuugaGcca........................   1     1    JCK-1
..............................................................................................................................................................................................................aagugccaugaacuugaucca........................   7     0    JT-2
..............................................................................................................................................................................................................aagugcAaugaacuugaucca........................   1     1    JCK-2
..............................................................................................................................................................................................................aaUugccaugaacuugaucca........................   10    1    JCK-1
..............................................................................................................................................................................................................aagugccaugaacuugauccU........................   1     1    LT-1
..............................................................................................................................................................................................................aaUugccaugaacuugaucca........................   19    1    JCK-2
..............................................................................................................................................................................................................aagugccaugaacuugauccC........................   1     1    JCK-2
..............................................................................................................................................................................................................aCgugccaugaacuugaucca........................   1     1    LT-2
..............................................................................................................................................................................................................aagugccaugaacuugauccU........................   2     1    JCK-2
..............................................................................................................................................................................................................aaNugccaugaacuugaucca........................   1     1    LT-2
..............................................................................................................................................................................................................aagugccaugaacuugauccU........................   2     1    LT-3
..............................................................................................................................................................................................................aagugccaugaacuugaucca........................   184   0    JCK-2
..............................................................................................................................................................................................................aagugccaugaacuugaucca........................   5     0    LCK-3
..............................................................................................................................................................................................................aagugccaugaacuugaucca........................   6     0    JT-3
..............................................................................................................................................................................................................Cagugccaugaacuugaucca........................   1     1    JCK-3
..............................................................................................................................................................................................................aagugccaugaacuugaucca........................   41    0    LT-3
..............................................................................................................................................................................................................aaguAccaugaacuugaucca........................   1     1    LT-2
..............................................................................................................................................................................................................aaUugccaugaacuugaucca........................   14    1    JCK-3
..............................................................................................................................................................................................................aNgugccaugaacuugaucca........................   1     1    JT-2
..............................................................................................................................................................................................................aagugccaugaacuugauccaA.......................   2     1    JCK-2
..............................................................................................................................................................................................................aagugccaugaacuugauccaU.......................   2     1    JCK-2
..............................................................................................................................................................................................................Cagugccaugaacuugauccag.......................   3     1    LT-1
..............................................................................................................................................................................................................aagugccaugaacuugaGccag.......................   1     1    JCK-1
..............................................................................................................................................................................................................aagugccaugaacuugauccag.......................   88    0    JCK-2
..............................................................................................................................................................................................................aagugccaugaacuugauccaA.......................   2     1    JCK-1
..............................................................................................................................................................................................................aagugccaugaacuugauccag.......................   17    0    LCK-3
..............................................................................................................................................................................................................aagugccaugaacuugauccag.......................   47    0    JCK-1
..............................................................................................................................................................................................................aagugccaugaacuugauccag.......................   29    0    LT-3
..............................................................................................................................................................................................................aagugccaugaacuugauccag.......................   27    0    LT-1
..............................................................................................................................................................................................................aagugccaugaacuugauccaU.......................   3     1    JCK-1
..............................................................................................................................................................................................................aagugccaugaacuugauccaA.......................   1     1    JCK-3
..............................................................................................................................................................................................................aagugccaugaacuugauccaA.......................   1     1    LT-3
..............................................................................................................................................................................................................aagugccaugaacuugauccag.......................   71    0    JCK-3
..............................................................................................................................................................................................................aagugccaugaacuugauccag.......................   2     0    LT-2
..............................................................................................................................................................................................................aagugccaugaacuugauccaU.......................   6     1    JCK-3
..............................................................................................................................................................................................................aagugccaugaacuugauccagC......................   1     1    LT-1
..............................................................................................................................................................................................................aagugccaugaacuugauccagU......................   2     1    LT-1
..............................................................................................................................................................................................................aagugccaugaacuugauccaga......................   2     0    LT-3
..............................................................................................................................................................................................................aagugccaugaacuugauccagU......................   1     1    JCK-3
..............................................................................................................................................................................................................aagugccaugaacuugauccaga......................   5     0    JCK-1
..............................................................................................................................................................................................................aagugccaugaacuugauccaga......................   4     0    JCK-3
..............................................................................................................................................................................................................aagugccaugaacuugauccagU......................   2     1    JCK-1
..............................................................................................................................................................................................................aagugccaugaacuugauccagC......................   1     1    JCK-2
..............................................................................................................................................................................................................aagugccaugaacuugauccaga......................   2     0    LT-1
```

Star / **Mature**

caauuagguuuagugaacagaagugccuuuuuuuuuuuuuuuuuuuugggguuaggaugcgacuauaaaaagcagaggaugguggggagaagaaauacgccacuuccaucuuguucguucacccucuguaucuuguucgauggcacuccccuuguauccaaucucucucucucucucucucucucucucucauauggugcgaguggaagugccaugaacuugauccagaaggugcucucgaacaaguuaca

...aagugccaugaacuugauccagU...	1	1	JCK-2
...aagugccaugaacuugauccaga...	11	0	JCK-2
...aagugccaugaacuugauccagC...	2	1	JCK-1
...aagugccaugaacuugauccaCa...	1	1	JCK-1
...aagugccaugaacuugauccaCa...	2	1	JCK-2
...aagugccaugaacuugauccaCa...	3	1	JCK-3
...aagugccaugaacuugauccagC...	1	1	JCK-3
...aagugccaugaacuugauccagaaggug...	1	0	JCK-3
...aagugccaugaacuugauccaAaaggug...	1	1	JCK-1
...agugccaugaacuugauc...	6	0	LCK-3
...agugccaugaacuugauc...	15	0	LT-2
...agugccaugaacuugauc...	7	0	LT-1
...agugccaugaacuugauc...	10	0	JCK-2
...agugccaugaacuugauc...	14	0	LT-3
...agugccaugaacuugauc...	5	0	JCK-1
...agugccaugaacuugauA...	1	1	LT-1
...agugccCugaacuugauc...	1	1	LT-2
...agugccaugaacuNgauc...	1	1	LCK-1
...agugccaugaacuugauU...	1	1	JCK-3
...agugccaugaacCugauc...	1	1	JCK-2
...agugccaugaacuugauc...	11	0	LCK-1
...agugccaugaacuugauc...	19	0	LCK-2
...agugccaugaacuugauc...	5	0	JCK-3
...agugccaugaacuugauU...	2	1	LT-3
...agugccaugaacuugaucc...	27	0	JCK-1
...agugccaugaacuugaucc...	47	0	LCK-2
...agugccaugaacuugaucc...	46	0	LT-3
...agugccaugaacuugaucc...	51	0	LT-2
...agugccaugaacuugaucc...	3	0	JT-2
...agugccaugaacuugaucc...	41	0	LCK-1
...aUugccaugaacuugaucc...	3	1	JCK-2
...agugccaugaacuugaucc...	6	0	JT-3
...agugccaugaacuugaucc...	45	0	LCK-3
...agugccaugaacuugaucc...	22	0	JCK-3
...aUugccaugaacuugaucc...	2	1	JCK-3
...agugccaugaacuugGucc...	1	1	LT-2
...agugccaugaacuugaucc...	30	0	JCK-2
...agugccaugaacuugaucc...	19	0	LT-1
...agugccaugaacuugaucU...	1	1	LT-1
...agugccaugaacuugaucA...	2	1	LT-2
...agugccaugaacuugaucca...	51	0	LCK-3
...agugccaugaacuugaucca...	22	0	LCK-2
...agugccaugaacuugaucca...	27	0	LT-2
...agugccaugaacuugaucca...	25	0	JCK-2
...agugccaugaacuugaucca...	5	0	JT-2
...aUugccaugaacuugaucca...	1	1	JCK-1
...agugccaugaacuugaucca...	10	0	JCK-1
...agugccaugaacuugCucca...	1	1	LT-2
...agugccaugaacuugaucca...	11	0	JCK-3
...aguAccaugaacuugaucca...	1	1	JCK-2
...aUugccaugaacuugaucca...	2	1	JCK-2
...agugccaugaacuugaucca...	18	0	LT-3
...Cgugccaugaacuugaucca...	1	1	LCK-3
...agugccaugaacuugaucca...	20	0	LCK-1
...agugccaugaacuugaucca...	20	0	LT-1
...agugccaugaacuugaucca...	12	0	JT-3
...aUugccaugaacuugaucca...	1	1	JCK-3
...agugccaugaacuugaCccag...	1	1	LT-3
...agugccaugaacuugauccag...	50	0	LT-1
...agugccaugaacuugauccaU...	3	1	JCK-2
...agugccaugaacuugauccCg...	1	1	JCK-2
...agugccaugaacuugauccag...	58	0	LT-3
...agugccaugaacuugauccag...	179	0	JCK-2
...agugccaugaacuugauccaA...	1	1	JCK-1
...Cgugccaugaacuugauccag...	1	1	JCK-3
...agugccaugaacuugauccag...	120	0	JCK-3
...agugccaugaacuugauccag...	28	0	LT-2
...aguAccaugaacuugauccag...	1	1	LT-3
...agugccaugaacuugauccaU...	4	1	JCK-1
...agugccGugaacuugauccag...	1	1	JCK-3

```
                                                                                                                           Star                                                                  Mature
caauuagguuuagugaacagaagugccuuuuuuuuuuuuuuuuuuugggguuaggaugcgacuauaaaaagcagaggaugguggggagaagaaauacgccacuuccaucuuguucguucacccucuguaucuuguucgauggcacuccccuuguauccaaucucucucucucucucucucucucucucucucauauggugcgaguggaagugccaugaacuugauccagaaggugcucucgaacaaguuaca
....................................................................................................................................................................................................................agugccaugaacuugauccaA.......................   1     1   JCK-3
....................................................................................................................................................................................................................aUugccaugaacuugauccag.......................   2     1   JCK-3
....................................................................................................................................................................................................................agugccaugaacuugauccag.......................   115   0   JCK-1
....................................................................................................................................................................................................................agugccaugaacuugauccaU.......................   4     1   JCK-3
....................................................................................................................................................................................................................agugccaugaacuugauccaA.......................   1     1   LT-1
....................................................................................................................................................................................................................agugccCugaacuugauccag.......................   1     1   JCK-2
....................................................................................................................................................................................................................aUugccaugaacuugauccag.......................   1     1   JCK-2
....................................................................................................................................................................................................................agugccaugaacuugauccaU.......................   2     1   LT-1
....................................................................................................................................................................................................................agugccaugaacuugauccag.......................   23    0   LCK-3
....................................................................................................................................................................................................................agugccaugaacuugauccaA.......................   3     1   JCK-2
....................................................................................................................................................................................................................agugccaugaacuugauccaAa......................   1     1   JCK-3
....................................................................................................................................................................................................................Cgugccaugaacuugauccaga......................   1     1   LT-1
....................................................................................................................................................................................................................agugccaugaacuugauccaga......................   2     0   LT-2
....................................................................................................................................................................................................................agugccaugaacuugauccaga......................   9     0   JCK-1
....................................................................................................................................................................................................................agugccaugaacuugauccaAa......................   1     1   JCK-2
....................................................................................................................................................................................................................Cgugccaugaacuugauccaga......................   2     1   JCK-3
....................................................................................................................................................................................................................agugccaugaacuugauccagU......................   4     1   JCK-2
....................................................................................................................................................................................................................Cgugccaugaacuugauccaga......................   4     1   JCK-2
....................................................................................................................................................................................................................agugccaugaacuugauccagU......................   2     1   JCK-3
....................................................................................................................................................................................................................agugccaugaacuugauccagU......................   6     1   JCK-1
....................................................................................................................................................................................................................agugccaugaacuugauccaga......................   6     0   LT-3
....................................................................................................................................................................................................................Ugugccaugaacuugauccaga......................   1     1   LT-3
....................................................................................................................................................................................................................agugccaugaacuugauccagC......................   1     1   JCK-2
....................................................................................................................................................................................................................agugccaugaacuugauccaga......................   2     0   LT-1
....................................................................................................................................................................................................................agugccaugaacuugauccaga......................   16    0   JCK-2
....................................................................................................................................................................................................................agugccaugaacuugauccaga......................   5     0   JCK-3
....................................................................................................................................................................................................................Cgugccaugaacuugauccaga......................   4     1   JCK-1
....................................................................................................................................................................................................................agugccaugaacuugauccagUa.....................   1     1   LT-1
.....................................................................................................................................................................................................................gugccaugaacuugaucc.........................   26    0   JCK-1
.....................................................................................................................................................................................................................Augccaugaacuugaucc.........................   1     1   LT-2
.....................................................................................................................................................................................................................gugccaugaacuugaucA.........................   8     1   JCK-2
.....................................................................................................................................................................................................................gugccaugaacuugaucA.........................   5     1   JCK-3
.....................................................................................................................................................................................................................Uugccaugaacuugaucc.........................   32    1   JCK-2
.....................................................................................................................................................................................................................Uugccaugaacuugaucc.........................   19    1   JCK-3
.....................................................................................................................................................................................................................gugccaugaacuugaucc.........................   69    0   LT-3
.....................................................................................................................................................................................................................gugccaugaacuugaucc.........................   11    0   JT-1
.....................................................................................................................................................................................................................Uugccaugaacuugaucc.........................   15    1   JCK-1
.....................................................................................................................................................................................................................gugccaugaacuugaucc.........................   40    0   LCK-2
.....................................................................................................................................................................................................................gugccaugaacuugaucc.........................   35    0   LT-2
.....................................................................................................................................................................................................................gugccaugaacuugaucc.........................   51    0   JCK-2
.....................................................................................................................................................................................................................gugccaugaacuugaucc.........................   40    0   JCK-3
.....................................................................................................................................................................................................................gugccaugaacuugaucc.........................   34    0   LT-1
.....................................................................................................................................................................................................................gugccaugaacuugaucc.........................   41    0   LCK-3
.....................................................................................................................................................................................................................Uugccaugaacuugaucc.........................   3     1   JT-1
.....................................................................................................................................................................................................................Augccaugaacuugaucc.........................   2     1   LCK-1
.....................................................................................................................................................................................................................gugccaugaacuugaucU.........................   1     1   LT-3
.....................................................................................................................................................................................................................gugccaugaacuugaucc.........................   34    0   LCK-1
.....................................................................................................................................................................................................................gugccaugaacuugaucca........................   14    0   JT-1
.....................................................................................................................................................................................................................gugccaugaacuugaucca........................   6     0   JCK-1
.....................................................................................................................................................................................................................Uugccaugaacuugaucca........................   5     1   JCK-1
.....................................................................................................................................................................................................................gugccaugaacuugaucca........................   11    0   JCK-2
.....................................................................................................................................................................................................................gugccaugaacuugaucca........................   22    0   LT-3
.....................................................................................................................................................................................................................gugccaugaacuugaucca........................   14    0   LT-1
.....................................................................................................................................................................................................................gugccaugaacuugaucca........................   6     0   JCK-3
.....................................................................................................................................................................................................................Uugccaugaacuugaucca........................   3     1   JCK-2
.....................................................................................................................................................................................................................gugccaugaacuugaucca........................   19    0   LCK-3
.....................................................................................................................................................................................................................gugccaugaacuugaucca........................   27    0   LT-2
.....................................................................................................................................................................................................................Uugccaugaacuugaucca........................   2     1   JCK-3
.....................................................................................................................................................................................................................gugccaugaacuugauccaA.......................   4     1   JT-2
.....................................................................................................................................................................................................................Uugccaugaacuugauccag.......................   79    1   JCK-2
.....................................................................................................................................................................................................................Augccaugaacuugauccag.......................   1     1   LCK-2
.....................................................................................................................................................................................................................gugccaugaacuugauccag.......................   16    0   JT-2
.....................................................................................................................................................................................................................Augccaugaacuugauccag.......................   1     1   JCK-2
.....................................................................................................................................................................................................................gugccaugaacuugauccaU.......................   6     1   LT-2
.....................................................................................................................................................................................................................gugccaugaacuugauccaA.......................   5     1   LT-3
.....................................................................................................................................................................................................................Uugccaugaacuugauccag.......................   1     1   LT-2
.....................................................................................................................................................................................................................gugccaugaacuugauccag.......................   46    0   LCK-1
.....................................................................................................................................................................................................................gugccaugaacuugauccaA.......................   4     1   JCK-3
.....................................................................................................................................................................................................................gugccaugaacuugauccaA.......................   3     1   LT-1
.....................................................................................................................................................................................................................gugccaugaacuugauccag.......................   63    0   LCK-3
```

Star / **Mature**

caauuagguuuagugaacagaagugccuuuuuuuuuuuuuuuuuuuuuuggguuaggaugcgacuauaaaaagcagaggaugguggggagaagaaauacgccacuuccaucuuguucguucacccucuguaucuuguucgauggcacucccuuguauccaaucucucucucucucucucucucucucucucucucauauggugcgaguggaagugccaugaacuugauccagaaggugcucucgaacaaguuaca

Read	Count	Mismatch	Sample
gugccaugaacuugauccag	56	0	LCK-2
gugccaugaacuugauccaU	2	1	JCK-3
Augccaugaacuugauccag	1	1	JT-3
gugccaugaCcuugauccag	1	1	LT-2
gugccaugaacuugauccag	84	0	LT-2
gugccaugaacuugauccaU	1	1	JCK-2
gugccaugaaAuugauccag	1	1	LT-2
gugccaugCacuugauccag	1	1	LT-1
gugccaugaacuugauccaA	3	1	JCK-1
gugccaugaacuugauccag	163	0	JCK-3
gugccaugaacuugauccaU	1	1	JCK-1
gugccaugaacuugauccag	103	0	LT-1
gugccaugaacCugauccag	1	1	LT-2
gugccaugaacuugauccaA	7	1	JT-3
gugccaugaacuugauccaA	8	1	JCK-2
gugccaugaacuugauccag	221	0	JCK-2
gugccaugaCcuugauccag	1	1	JCK-2
Uugccaugaacuugauccag	66	1	JCK-3
gugccaugaacuugauccag	26	0	JT-3
Uugccaugaacuugauccag	6	1	JT-2
Uugccaugaacuugauccag	34	1	JCK-1
gugccaugaacuugauccag	47	0	JT-1
Uugccaugaacuugauccag	20	1	JT-1
gugccaugaCcuugauccag	1	1	JT-3
gugccaugaacuugauccag	125	0	LT-3
Uugccaugaacuugauccag	1	1	LT-3
Uugccaugaacuugauccag	10	1	JT-3
gugccaugaacuugauccag	138	0	JCK-1
gugccaugaacuugCuccag	1	1	LT-3
gugccaugaacuugCuccaga	1	1	JCK-1
gugccGugaacuugauccaga	1	1	JCK-1
gugccaugaacuugauccaga	649	0	LT-3
Cugccaugaacuugauccaga	1	1	JCK-1
gugccaugaacuugauccagC	1	1	JCK-2
gugccaugaacuugauccagU	8	1	JCK-2
gNgccaugaacuugauccaga	1	1	JCK-3
gugccaugGacuugauccaga	1	1	JCK-1
gugccaugaacuugauccaga	444	0	LT-1
gugccaugaacuugauccaga	1669	0	JCK-1
gugccaugaacuugauccaga	90	0	LCK-1
gugccaugaacuugauccaAa	2	1	LT-2
gugccaugaacuugauccaAa	5	1	LT-1
gugccaugaacuugauccagU	10	1	JCK-3
gugccaugaacuugaucCga	1	1	LCK-3
gugccaugaacuugauccaAa	4	1	JCK-2
gugccaugaacuugauccaUa	1	1	LT-3
gugccaugaacNugauccaga	7	1	JCK-1
gugccaugaacuugauccaCa	1	1	JCK-1
guNccaugaacuugauccaga	1	1	LT-2
gNgccaugaacuugauccaga	3	1	JCK-2
gugccaugaacuugaucUaga	2	1	JCK-1
gugccaugaacuugaucCga	1	1	LT-1
gugccaugaacuugCuccaga	1	1	LT-2
Augccaugaacuugauccaga	7	1	JCK-3
gugccCugaacuugauccaga	1	1	LT-3
gugccaugaacuugaucCga	2	1	JCK-1
gugccCugaacuugauccaga	2	1	JCK-3
gugccaugaacuugauccaUa	1	1	JCK-2
gugccaugaacuugauccaAa	7	1	LT-3
gugccaugaacuugauccaAa	9	1	JCK-3
gugccaugaacuuUauccaga	1	1	JCK-1
gugccaugaacuugauccagC	1	1	LT-2
gugccCugaacuugauccaga	4	1	JCK-2
gugccCugaacuugauccaga	2	1	LT-2
Uugccaugaacuugauccaga	1	1	JT-1
gugccaugaacuugauccaga	134	0	JT-1
gugccaugaacuugauccagU	6	1	JCK-1
gugccaugaacuugauccagU	16	1	LCK-1
gugccCugaacuugauccaga	1	1	JT-1
gugccaugaacuugCuccaga	2	1	LT-3

Star Mature

caauuagguuuagugaacagaagugccuuuuuuuuuuuuuuuuuuuugggguuaggaugcgacuauaaaaagcagaggaugguggg agaagaaauacgccacuuccaucuuguucguucacccucuguaucuuguucgauggcacuccccuuguauccaaucucucucucucucucucucucucucucucauauggugcgaguggaagugccaugaacuugauccagaaggugcucucgaacaaguuaca

guAccaugaacuugauccaga	2	1	JCK-2
Uugccaugaacuugauccaga	4	1	JT-2
gugcUaugaacuugauccaga	2	1	JCK-2
gugUcaugaacuugauccaga	2	1	JCK-3
gugccaugCacuugauccaga	1	1	JCK-2
Uugccaugaacuugauccaga	6	1	JT-3
gugccaugaacuugauccaga	2129	0	JCK-3
gugccaugaacuugauccagU	25	1	LCK-2
gugccaugaacuugauccagG	1	1	JCK-2
Uugccaugaacuugauccaga	666	1	JCK-1
Augccaugaacuugauccaga	3	1	LT-3
Uugccaugaacuugauccaga	871	1	JCK-3
gugccaugaacuugauccaAa	4	1	JCK-1
gugccaugaacuugauccaga	295	0	LCK-3
gugccaugaacuCgauccaga	1	1	JCK-3
gugccaugaacuugauccaga	107	0	LCK-2
gugcAaugaacuugauccaga	1	1	JT-1
Cugccaugaacuugauccaga	4	1	JCK-2
gugccaugaacuugauccagU	9	1	JT-1
gugccaugaacuugauccaNa	3	1	JCK-1
gugccaugaacCugauccaga	1	1	JCK-1
Cugccaugaacuugauccaga	1	1	LT-3
Augccaugaacuugauccaga	4	1	JCK-2
gugccaugaacuCgauccaga	1	1	JCK-2
gugccaugaCcuugauccaga	2	1	JCK-1
gugccaugaacuugauccagC	7	1	LT-3
gugcUaugaacuugauccaga	3	1	JCK-3
gugccaugaacuugauccagU	9	1	LT-2
gugccaugaacuugauccaUa	2	1	LT-1
gugccNugaacuugauccaga	1	1	LCK-1
gugccaugaacuugaucGaga	1	1	JCK-3
gugccaugaacuugCuccaga	1	1	LT-1
gugUcaugaacuugauccaga	2	1	JCK-2
gugccaugaacuugCuccaga	2	1	JCK-2
gugccaugaacuugauccagU	22	1	LT-3
gugccaugaacuugauccaga	463	0	LT-2
gugccaugaacuugauccNga	1	1	JCK-1
gugccaugaacuugauccCga	1	1	LT-3
gCgccaugaacuugauccaga	2	1	JCK-3
gugccaugaacuugauccaAa	1	1	LCK-3
gugccaugaacuugauccaga	2906	0	JCK-2
gugccaugaacuugauccCga	1	1	JT-1
Uugccaugaacuugauccaga	1	1	LCK-3
gugccaugCacuugauccaga	2	1	LT-2
gugccCugaacuugauccaga	2	1	LT-1
gugccaugaacuugaucGaga	2	1	JCK-1
gugccaugCacuugauccaga	1	1	JCK-3
gugccaugaacuCgauccaga	1	1	JCK-1
gugccaugaacuugauccGga	1	1	JCK-3
guAccaugaacuugauccaga	1	1	JCK-3
Uugccaugaacuugauccaga	1141	1	JCK-2
gugccaugaacuugauccaga	56	0	JT-2
gugccaugaacuugauccagU	5	1	LT-1
gNgccaugaacuugauccaga	2	1	JCK-1
gugccaugaacuugauccaga	43	0	JT-3
gugccaugaacuugauccCga	1	1	LCK-1
gugccaugaCcuugauccaga	2	1	JCK-2
Augccaugaacuugauccaga	1	1	LT-1
gugccaugaacuugauccagC	2	1	LT-1
Cugccaugaacuugauccaga	4	1	JCK-3
gugccaugaacNugauccaga	1	1	JT-1
gugcAaugaacuugauccaga	2	1	JCK-1
Augccaugaacuugauccaga	3	1	JCK-1
gugccCugaacuugauccaga	4	1	JCK-1
gugccaugNacuugauccaga	1	1	JCK-3
gCgccaugaacuugauccaga	1	1	LT-3
gugccaugaCcuugauccaga	1	1	LCK-3
gugccaugaacuugauccagaC	2	1	JCK-1
gugccaugaacuugauccagaa	4	0	JCK-1
gugccaugaacuugauccaAaa	1	1	JCK-2

Star   Mature

caauuagguuuagugaacagaagugccuuuuuuuuuuuuuuuuuuuggguuaggaugcgacuauaaaaagcagaggauggugggagaagaaauacgccacuuccaucuuguucguucacccucuguaucuuguucgauggcacuccccuuguauccaaucucucucucucucucucucucucucucucucucauauggugcgaguggaagugccaugaacuugauccagaaggugcucucgaacaaguuaca

Uugccaugaacuugauccagaa	4	1	JCK-3
gugccaugaacuugauccagaU	2	1	JCK-3
gugccaugaacuugauccagaC	4	1	LT-1
Uugccaugaacuugauccagaa	2	1	JCK-2
gugccaugaacuugauccagaa	5	0	LT-3
gugccaugaacuugauccagaa	11	0	LCK-3
gugccaugaacuugauccagaa	8	0	JCK-2
gugccaugaacuugauccagaU	3	1	JCK-2
Uugccaugaacuugauccagaa	1	1	JCK-1
gugccaugaacuugauccagaU	1	1	LT-1
gugccaugaacuugauccagaC	3	1	LT-3
gugccaugaacuugauccagaU	3	1	JCK-1
gugccaugaacuugauccagaC	4	1	LT-2
gugccaugaacuugauccagaC	2	1	JCK-2
gugccaugaacuugauccagaa	9	0	JCK-3
gugccaugaacuugauccagaa	2	0	LT-1
gugccaugaacuugauccagaaA	3	1	JCK-3
gugccaugaacuugauccagaaA	8	1	JCK-2
gugccaugaacuugauccagaaA	3	1	JCK-1
gugccaugaacuugauccagaagguU	2	1	JCK-3
ugccaugaacuugaucca	1	0	LT-1
ugccaugaacuugaucca	8	0	LCK-3
ugccaugaacuugaCcca	1	1	LT-3
ugccaugaacuugaucca	1	0	JCK-3
ugccaugaacuugaucca	7	0	JCK-2
ugccaugaacuugaucca	1	0	JCK-1
ugccaugaacuugaucca	6	0	LT-3
ugccaugaacuugaucca	7	0	LT-2
ugccaugaacuugauccaA	2	1	JCK-3
ugccaugaacuugauccCg	1	1	JCK-2
ugccaugaacuugauccaA	1	1	JCK-1
ugccaugaacuugauccag	43	0	JT-3
ugccaugaacuugauccaA	1	1	JCK-2
ugccaugaacuugauccag	101	0	JCK-1
uNccaugaacuugauccag	1	1	JCK-1
ugccaugaacuugauccaU	1	1	LT-3
ugccaugaacuugauccaU	1	1	JCK-1
ugccaugaacuugauccCg	1	1	LT-2
ugccaugaacuugauccag	9	0	JT-1
ugccaugaCcuugauccag	1	1	LT-1
ugccaugaacuugauccag	26	0	JT-2
ugccaugaCcuugauccag	1	1	LT-3
ugccaugCacuugauccag	1	1	LT-1
ugccaugaacuugauccag	102	0	JCK-3
ugccaugaacuugauccag	259	0	LT-3
ugccaugaacuugauccag	167	0	JCK-2
ugccaugaacuugauccag	114	0	LT-2
Cgccaugaacuugauccag	1	1	JCK-1
ugccaCgaacuugauccag	1	1	JCK-2
Cgccaugaacuugauccag	2	1	JCK-3
ugccaugaacuugauccag	161	0	LT-1
Cgccaugaacuugauccag	1	1	JCK-2
ugccaugaCcuugauccag	1	1	LT-2
ugccaugCacuugauccag	1	1	JCK-3
ugccauCaacuugauccag	1	1	JCK-1
ugccaugaacuugauccag	74	0	LCK-3
ugccGugaacuugauccaga	1	1	LT-1
ugccaugaacuugauccaAa	1	1	JCK-3
ugccaugaacuugauccaga	12	0	JT-3
ugccaugGacuugauccaga	1	1	JCK-2
ugccCugaacuugauccaga	1	1	JCK-2
uNccaugaacuugauccaga	2	1	JCK-1
ugccaugaacuugauccagU	9	1	LT-2
ugNcaugaacuugauccaga	1	1	LT-2
ugccaugaacuugauccCga	3	1	JCK-2
ugccaugaacuugauccaga	86	0	LCK-2
ugccaugCacuugauccaga	1	1	JT-1
ugccaugaacuugauccaga	184	0	LCK-3
ugccaugaacuugauccaNa	1	1	JCK-1
ugccaugaacuugauccaga	476	0	LT-1

Star / Mature

caauuagguuuagugaacagaagugccuuuuuuuuuuuuuuuuuuuggguuaggaugcgacuauaaaaagcagaggaugguggggagaagaaauacgccacuuccaucuuguucguucacccuc**uguaucuuguucgauggcacuc**cccuuguauccaaucucucucucucucucucucucucucucauaugguggcgaguggaa**gugccaugaacuugauccaga**aggugcucucgaacaaguuaca			
Cgccaugaacuugauccaga	1	1	LCK-3
uAccaugaacuugauccaga	1	1	JCK-2
ugccaugaacuugauNcaga	1	1	JCK-3
uAccaugaacuugauccaga	1	1	LCK-3
ugcGaugaacuugauccaga	2	1	JCK-1
ugccaugaacuugauccagC	2	1	JCK-3
ugccaugaacuugauccaga	60	0	LCK-1
ugccaugaacuugauccaga	93	0	JT-1
ugccaugaacuugauccaga	1134	0	JCK-3
ugccCugaacuugauccaga	1	1	LT-3
ugccaugaacuNgauccaga	1	1	JCK-1
ugccaugaCcuugauccaga	1	1	JCK-2
ugccaugaacuugCuccaga	1	1	LCK-3
ugccaugaCcuugauccaga	1	1	JCK-1
ugccCugaacuugauccaga	1	1	LT-1
ugccaugaacuugauccagU	4	1	LT-3
ugccaugaacuuUauccaga	1	1	JCK-2
ugccaugaacuugauccaga	6	0	JT-2
ugccauAaacuugauccaga	1	1	LT-3
ugccaugaacuCgauccaga	1	1	JCK-3
ugccaugaacuuUauccaga	1	1	JCK-3
ugccaugCacuugauccaga	1	1	LT-2
uAccaugaacuugauccaga	2	1	LT-1
ugccaugaacuugauccagU	1	1	JCK-1
ugccaugaacuugauccagG	1	1	JCK-1
uNccaugaacuugauccaga	2	1	JCK-3
ugccaugaCcuugauccaga	4	1	JCK-3
ugccaugaacuugauccagC	3	1	JCK-2
ugccaugaacuugauccCga	2	1	JCK-1
ugccCugaacuugauccaga	1	1	LT-2
ugccaugaacuugCuccaga	1	1	LT-3
ugccaugaacuugauccaga	848	0	JCK-1
ugcAaugaacuugauccaga	1	1	JCK-2
ugccaugaacuugauccaga	290	0	LT-2
ugccaugCacuugauccaga	1	1	LT-3
ugccaugaacuugauccaga	657	0	LT-3
ugccaugaacuugauccagC	4	1	LT-3
ugccaugaacuugauccagG	1	1	JCK-2
ugccaugaacuugaCccaga	1	1	JCK-3
ugccaugaacuugauccaga	1507	0	JCK-2
ugccaugaacuugCuccaga	1	1	JT-1
ugccCugaacuugauccaga	4	1	JCK-3
ugccaugaacuugauccagU	5	1	JCK-2
ugccaugCacuugauccaga	1	1	LCK-3
ugccaugaacuugauccagU	7	1	JCK-3
ugcAaugaacuugauccaga	1	1	JCK-1
ugccaugaacuugauccagG	1	1	LT-2
ugccaugaCcuugauccaga	1	1	LT-1
ugccaugaacuugauccagN	1	1	JCK-1
uUccaugaacuugauccaga	1	1	JCK-3
ugccaugaacuugauccagU	3	1	LT-1
ugccaugaacuugauccagaa	5	0	JCK-1
ugccaugaacuugauccagaU	10	1	LT-1
ugccaugaacuugauccagaU	2	1	JT-3
ugccaugaacuugauccagaa	3	0	JCK-3
ugccaugaacuugauccagaU	5	1	LCK-3
ugccaugaacuugauccagaU	1	1	JCK-1
ugccaugaacuugauccagaa	1	0	JCK-2
ugccaugaacuugauccagaC	5	1	LT-3
ugccaugaacuugauccagaU	9	1	LT-3
ugccaugaacuugauccagaC	3	1	LT-1
ugccaugaacuugauccagaU	5	1	LT-2
ugccaugaacuugauccagaa	4	0	LT-2
ugccaugaacuugauccagaU	2	1	JCK-2
ugccaugaacuugauccagaag	2	0	JCK-3
ugccaugaacuugauccagaag	1	0	JCK-2
ugccaugaacuugauccagaagg	1	0	JCK-2
ugccaugaacuugauccagaagg	1	0	JCK-1
ugccaugaacuugauccagaagg	5	0	LT-3
ugccaugaacuugauccagaagg	3	0	JCK-3

Star Mature

```
caauuagguuuagugaacagaagugccuuuuuuuuuuuuuuuuuuuuggguuaggaugcgacuauaaaaagcagaggauggugggagaagaaauacgccacuuccaucuuguucguucacccucuguaucuuguucgauggcacuccccuuguauccaaucucucucucucucucucucucucucucucucauauggugcgaguggaagugccaugaacuugauccagaaggugcucucgaacaaguuaca
..................................................................................................................................................................................................................gccaugaacuugauccag.......................   1   0   JCK-2
..................................................................................................................................................................................................................gccaugaacuugauccaga......................   1   0   JCK-3
..................................................................................................................................................................................................................gccaugaacuugauccaga......................   1   0   JCK-1
...................................................................................................................................................................................................................ccaugaacuugauccagU......................   1   1   JCK-3
...................................................................................................................................................................................................................ccaugaacuugauccagU......................   4   1   LCK-3
...................................................................................................................................................................................................................ccaugaacuugauccaga......................   2   0   LT-3
...................................................................................................................................................................................................................ccaugaacuugauccagU......................   2   1   JCK-1
...................................................................................................................................................................................................................ccaugaacuugauccaga......................   1   0   JCK-2
...................................................................................................................................................................................................................ccaugaacuugauccagaaggu..................   1   0   LT-3
........................................................................................................................................................................................................................aacuugauccagaaggugcucu.............   1   0   JCK-1
........................................................................................................................................................................................................................aacuugauccagaaggugcucu.............   1   0   JCK-2
.........................................................................................................................................................................................................................acuugauccagaaggugcucucga..........   1   0   LT-2
...............................................................................................................................................................................................................................uccagaaggugcucucgaacaaguuac.   1   0   LT-2
.................................................................................................................................................................................................................................cagaaggugcucucgaacaagu....   7   0   LCK-3
.................................................................................................................................................................................................................................cagaaggugcucucgaacaagu....   2   0   JCK-1
.................................................................................................................................................................................................................................cagaaggugcucucgaacaagu....   4   0   JCK-3
.................................................................................................................................................................................................................................cagaaggugcucucgaacaagu....   3   0   LT-3
.................................................................................................................................................................................................................................cagaaggugcucucgaacaagu....   2   0   LT-1
.................................................................................................................................................................................................................................cagaaggugcucucgaacaagu....   4   0   JCK-2
..................................................................................................................................................................................................................................agaaggugcucucgaacaagu....   6   0   LCK-3
..................................................................................................................................................................................................................................agaaggugcucucgaacaagu....   7   0   LT-3
..................................................................................................................................................................................................................................agaaggugcucucgaacaagu....   3   0   JCK-2
..................................................................................................................................................................................................................................agaaggugcucucgaacaagu....   1   0   LT-1
..................................................................................................................................................................................................................................agaaggugcucucgaacaNgu....   1   1   JCK-1
..................................................................................................................................................................................................................................agaaggugcucucgaacaagu....   1   0   JCK-3
..................................................................................................................................................................................................................................agaaggugcucucgaacaaguu...   3   0   JCK-1
..................................................................................................................................................................................................................................agaaggugcucucgaacaaguu...   1   0   JCK-3
..................................................................................................................................................................................................................................agaaggugcucucgaacaaguu...   1   0   LT-1
..................................................................................................................................................................................................................................agaaggugcucucgaacaaguu...   2   0   LT-3
..................................................................................................................................................................................................................................agaaggugcucucgaacaaguuaca   1   0   LT-3
...................................................................................................................................................................................................................................gaaggugcucucgaacaaguua..   3   0   LT-1
...................................................................................................................................................................................................................................gaaggugcucucgaacaaguuac.   1   0   JCK-2
...................................................................................................................................................................................................................................gaaggugcucucgaacaaguuac.   2   0   JCK-3
.....................................................................................................................................................................................................................................aggugcucucgaacaagu....   1   0   LT-3
.....................................................................................................................................................................................................................................aggugcucucgaacaaguA...   1   1   LT-1
.....................................................................................................................................................................................................................................aggugcucucgaacaaguuaca   1   0   LT-2
.....................................................................................................................................................................................................................................aggugcucucgaacaaguuacC   1   1   LT-3
```
